# Corneal higher-order aberration changes after accelerated cross-linking for keratoconus

**DOI:** 10.1186/s12886-022-02457-0

**Published:** 2022-05-18

**Authors:** Abdelrahman Salman, Marwan Ghabra, Taym R. Darwish, Obeda Kailani, Hussein Ibrahim, Hakam Ghabra

**Affiliations:** 1 Tratous Specialist Eye Center, Tishreen University, P.O.Box:25, Latakia, Syria; 2 Whipps Cross University Hospital, Leytonstone, London, UK; 3 Department of Ophthalmology, Tishreen University, Latakia, Syria; 4 AKC FRCOphth CertLRS Queen Mary’s Hospital, King’s College Hospital NHS Foundation Trust, London, UK; 5 Surgical Eye Hospital, Health Ministry, Damascus, Syria; 6 Harley Street Eye Centre, London, UK

**Keywords:** Keratoconus, Corneal cross-linking, Sirius, Higher order aberrations, Scheimpflug, Topography, Visual acuity

## Abstract

**Aim:**

To evaluate changes in corneal higher-order aberrations (HOAs) following epithelium-off accelerated corneal cross-linking (A-CXL) and to explore the impact on visual acuity.

**Methods:**

In this retrospective case series, 32 eyes of 24 patients with keratoconus (KC) underwent A-CXL. Treatment was delivered at 10 mW/cm^2^ for 9 min with a total dose of 5.4 J/cm^2^. The following anterior corneal HOAs: total corneal HOAs, trefoil, secondary trefoil, coma, secondary coma, secondary astigmatism and spherical aberrations were analysed using the Scheimpflug-Placido Sirius (CSO, Italy) corneal topographer at baseline and 12 months following treatment. Multivariate analysis was used to evaluate the independent effect of HOA subtypes on changes in uncorrected distance visual acuity (UDVA) and corrected distance visual acuity (CDVA).

**Results:**

At one year post CXL, UDVA and CDVA were significantly improved, -0.13 ± 0.19 LogMAR (*P* = 0.0005) and -0.08 ± .0.11 LogMAR (*P* = 0.0003), respectively. The mean preoperative trefoil, secondary trefoil, secondary coma and secondary astigmatism were 0.95 ± 0.46; µm, 0.20 ± 0.11; µm, 0.29 ± 0.19; µm and 0.42 ± 0.17 µm, respectively. At one year, the mean values decreased significantly to 0.77 ± 0.47 µm, 0.15 ± 0.11 µm, 0.25 ± 0.18 µm and 0.34 ± 0.18 µm, respectively (*P* < 0.05, for all). No independent relationship between any HOA changes and change in UDVA was observed. A reduction in secondary coma aberration was associated with a change in CDVA (95% CI 0.01–1.34, *P* = 0.048; β = 0.67).

**Conclusion:**

A 9-min protocol of Accelerated corneal cross-linking is an effective treatment in improving corneal HOAs at 12 months follow up, in eyes with progressive keratoconus at one year follow-up. A change in secondary coma had a statistically significant and independent effect on CDVA.

## Introduction

Keratoconus (KC) is a progressive corneal disease characterized by thinning of the central or para-central portion of the cornea resulting in irregular astigmatism and visual deterioration [1]. The aetiology and pathogenesis of corneal ectasia is complex and multiple factors including biomechanics, enzymology, proteomics, and molecular genetics have been reported to impact progression and severity [2]. Although, the complete aetiology of keratoconus is not fully understood, focal thinning and resultant biomechanical weakening, independent of other factors, has been established as a predictor of ectasia and subsequent cone formation [3]. This may suggest that focal weakening is likely to be the final stage in what is a multifactorial condition. Corneal thinning and corneal irregularities may induce significant amounts of higher-order aberrations (HOAs) which differ significantly from the aberrations of a normal cornea [4].

Corneal cross-linking (CXL) was first introduced nearly 20 years ago to halt disease progression of keratoconus [5]. The procedure aims to increase the rate of formation of chemical bonds between the fibres of corneal collagen by means of a highly localized photopolymerization using ultraviolet A (UVA) light and a photosensitizer, riboflavin [6]. CXL can also have beneficial visual, topographic and aberrometric effects [7–9]. Improvements in uncorrected distance visual acuity (UDVA), corrected distance visual acuity (CDVA), as well as maximum keratometry and several topographic indices following CXL have been reported [10]. Interpreting the changes in corneal HOAs after corneal cross-linking is useful to establish the impact of the interventions on optical functions and visual outcomes. Several studies have evaluated the change in corneal HOAs after A-CXL [7–10], to our knowledge this is the first study designed to evaluate the effect of changes in corneal HOAs on visual acuity after a 9-min A-CXL protocol.

This study was designed to evaluate the effect of a novel 9-min accelerated CXL (A-CXL) protocol on anterior corneal HOAs and to establish the impact on functional visual outcomes (UDVA and CDVA) in patients with progressive KC.

### Patients and methods

This was a retrospective case series of patients with progressive keratoconus undergoing an accelerated collagen crosslinking protocol of 10 mW/cm^2^ for 9 min. Treatment was conducted at Tishreen University Hospital. This study adhered to the tenets of the Declaration of Helsinki and was approved by the Research and Ethics Committee of Tishreen University Hospital. Prior to treatment, written informed consent was obtained from all patients.

Patients aged 18 years and older at the time of treatment with demonstrated progressive KC on serial visits with 12-month post-operative keratometric and topographic data were included in the study. A diagnosis of KC was established in concordance with the Global Consensus on Keratoconus and Corneal Ectatic Diseases Report [11]. Progression of keratoconus was defined as: at least 1 diopter increase in the anterior maximum keratometry or in the manifest refraction spherical equivalent (MRSE), a decrease of 5% in the minimum pachymetry, or loss of at least two lines of corrected distance visual acuity over 12 months [12].

Exclusion criteria included breastfeeding or pregnancy, central or paracentral corneal opacities or scarring, uncontrolled stromal or ocular surface disease (viral / autoimmune), previous ocular surgery, and a corneal thickness of less than 400 µm. Patients who developed postoperative scarring or corneal haze were also excluded from the analysis due to the secondary optical aberrations that result with both scarring and haze [13].

All keratoconic eyes were classified according to Amsler-Krumeich classification [14]. Contact lens-wearing patients were asked to discontinue wearing their lenses for 3-weeks and 1-week for rigid and soft contact lenses, respectively. Evaluation included the measurement of LogMAR visual acuity (UDVA, CDVA), manifest refraction spherical equivalent (MRSE), slit-lamp biomicroscopy, retinoscopy and fundoscopy. Topography, tomography and aberrometry data were measured using the Sirius Scheimpflug-Placido topographer (Costruzioni Strumenti Oftalmici, Florence, Italy). Anterior corneal HOAs were measured at the central 6 mm zone. Three serial images were acquired to ensure reproducibility, centration, alignment and focus for both eyes. Patients were asked to blink before each image capture to eliminate the effect of corneal surface dryness and optimise image acquisition. Seventeen continuous mires on Placido disc imaging, were a requirement for all patients, ensuring optimal videokeratography acquisition and Zernike coefficient calculation for a 6 mm simulated pupil. Data acquisition was uniform for all data points for consistency (CSO, Phoenix v.2.6).

### Surgical procedure

An epithelium-off A-CXL technique was performed in all subjects. Eyes were anaesthetized using topical instillation of Proparacaine Hydrochloride 0.5% (Proparacaine Rama 0.5%, Rama Pharma, Syria) eye drops administered at 2-min intervals, for a duration of 10-min preoperatively. Topical povidone-iodine 5% was used for sterilization. The central 8–9 mm corneal epithelium was removed manually using a blunt spatula and dry sponge, without alcohol assistance. Ultrasonic pachymetry was performed to ensure a minimum corneal thickness of 400 μm prior to ultraviolet A (UVA) exposure. Twenty minutes prior to irradiance, Riboflavin with Dextran (0.1% Riboflavin in 20% Dextran. Medio Cross, Germany) solution was applied every 2 min. The saturation of the anterior chamber with riboflavin was checked with slit-lamp biomicroscopy. The 32 eyes were irradiated with the Vega C.B.M-X Linker (CSO, Italy) using the A-CXL 10 mW/cm^2^ UVA for 9 min to achieve a total energy of 5.4 J/cm^2^. During the 9 min of irradiance, riboflavin solution was applied every 2 min. At the end of the procedure, the corneal surface was irrigated with balanced salt solution (BSS) and a soft contact lens was applied for 5 days. Topical moxifloxacin 0.5% (Megamox, Rama Pharma, Syria) and fluorometholone 0.1% (Methouflor 0.1%, Diamond Pharma, Syria) eye drops were prescribed for 1 week and 2 weeks, respectively. The detailed surgical protocol utilized for A-CXL has been described by the group previously [10, 15].

### Mean outcomes measures

Measurements UDVA, CDVA, MRSE, maximum keratometry, mean keratometry, simulated topographic cylinder, symmetry index front (SIf), total Baiocchi-Calossi-Versaci index (BCV) and minimum corneal thickness (ThkMin). Anterior corneal HOAs were collected from the Sirius at the central 6 mm zone. The Sirius Scheimpflug-Placido topographer provides corneal aberrometry utilizing elevation map algorithms. Normalized coefficients were used, expressed in microns of wavefront error, root mean square (RMS), and labeled with International Organization for Standardization (ISO) standardized double-index Zernike symbols [16]. The collected HOAs data included: Root mean square (RMS) total HOAs, RMS trefoil Z (3, ± 3), RMS trefoil II Z (5, ± 3), RMS coma Z (3, ± 1), RMS coma II Z (5, ± 1), RMS astigmatism II Z (4, ± 2), and RMS spherical aberration I Z (4, 0). These measurements were obtained at baseline and at 12 months after treatment.

### Statistical analysis

Visual acuity was converted to a logMAR notation. A paired t-test was used to test the statistical significance between HOAs at baseline and HOAs 1 year after A-CXL. Multivariate regression analysis was used to identify the factors associated with changes in UDVA and CDVA. analysis was performed using generalized estimating equations to correct for patients in whom both eyes were included in the dataset. Analyses were performed using SPSS software (version 21.0, International Business Machines Corp.). A *p*-value of less than 0.05 was considered significant.

## Results

A total of 32 eyes of 24 patients were included in the study. The mean age was 24.7 ± 6.7 years. 16 (66.67%) patients were females. According to the Amsler-Krumeich classification, 24 eyes had stage I KC and 8 eyes had stage II KC. All 32 eyes had central KC, with an apex within a central 2 mm radius.

### Visual, refractive and topographic outcomes

LogMAR UDVA and CDVA were significantly improved (-0.13 ± 0.19 and -0.08 ± 0.11 respectively, *P* < 0.05 for both) at one year postoperatively. The mean values of MRSE, sphere and manifest astigmatism were significantly improved, 0.52 ± 0.63 D, 0.39 ± 0.61 D and 0.26 ± 0.46 D, respectively (*P* < 0.05 for all). Mean keratometry, simulated cylinder, SIf and BCV did not show a statistically significant difference from baseline at 12 months (*P* > 0.05 for all). Maximum keratometry significantly decreased from baseline line values from 54.62 ± 4.13 D to 54.0 ± 4.63 D at one year post A-CXL. The mean values of ThkMin were significantly decreased (-11.09 ± 19.60 µm, *P* < 0.05). Table 1 shows the visual, refractive and topographic outcomes. At 1 year-follow-up, one eye (3.12%) lost 1 Snellen line of the CDVA, 14 (43.75%) had no change and 17 (53.13%) eyes gained one line or more. The maximum keratometry was decreased or unchanged in 29 eyes and increased by more than 1 D in 3 eyes.

**Table 1:** Patients characteristics at baseline and at 1 year after cross-linking

	Preoperative		Postoperative		Change		
	Mean	Std. Dev	Mean	Std. Dev	Mean	Std. Dev	*p* value
LogMAR UDVA	0.63	0.43	0.50	0.40	-0.13	0.19	**0.0005**
LogMAR CDVA	0.30	0.25	0.22	0.20	-0.08	0.11	**0.0003**
MRSE (D)	-2.64	2.45	-2.12	2.29	0.52	0.63	**0.0001**
Sphere (D)	-1.03	2.42	-0.64	2.22	0.39	0.61	**0.001**
Cylinder (D)	-3.21	1.46	-2.95	1.37	0.26	0.46	**0.0032**
Mean keratometry (D)	46.78	2.00	46.81	2.37	0.03	1.85	0.9296
Maximum Keratometry (D)	54.62	4.13	54.00	4.63	0.61	1.41	**0.029**
Simulated Cylinder (D)	-3.32	1.68	-3.47	2.16	-0.15	0.90	0.3484
SIf (D)	5.84	3.02	5.45	3.19	-0.39	1.33	0.1072
BCV (D)	3.01	2.44	2.51	1.26	-0.50	2.25	0.2109
ThkMin (µm)	432.19	27.04	421.09	26.42	-11.09	19.60	**0.0032**

*UDVA* Uncorrected distance visual acuity, *CDVA* Corrected distance visual acuity, *MRSE* Manifest refraction spherical equivalent, *D* Diopter, *SIf* Symmetry index front, *BCV* Baiocchi-Calossi-Versaci, *ThkMin* Minimum corneal thickness, *µm* Micron. P. Chi-squared test. Statistically significant values (*P* < 0.05). Values in bold are statistically significant

### Change in higher-order aberrations

Total HOAs, coma and spherical aberration values showed no statistically significant difference from baseline at 12 months post A-CXL (*P* > 0.05 for all). However, trefoil, trefoil II, coma II and astigmatism II decreased significantly at one year compared to baseline values (*P* < 0.05 for all). Table 2 shows the anterior corneal aberrations changes in the study population.

**Table 2:** Changes in anterior corneal HOAs one year after A-CXL

	Preoperative		Postoperative		Change		
	Mean	Std. Dev	Mean	Std. Dev	Mean	Std. Dev	*p* value
Total HOAs	2.40	1.28	2.24	1.17	-0.16	0.55	0.1032
Z (3, ± 3) trefoil	0.95	0.46	0.77	0.47	-0.18	0.28	**0.0014**
Z (5, ± 3) trefoil II	0.20	0.11	0.15	0.11	-0.05	0.12	**0.0356**
Z (3, ± 1) coma	2.10	1.13	1.95	1.22	-0.14	0.46	0.0846
Z (5, ± 1) coma II	0.29	0.19	0.25	0.18	-0.05	0.10	**0.0113**
Z (4, ± 2) astigmatism II	0.42	0.17	0.34	0.18	-0.08	0.15	**0.0056**
Z (4, 0) spherical aberration I	0.10	0.35	0.07	0.32	-0.03	0.17	0.302

*HOAs* Higher-order aberrations, *Z* Zernike. P. Chi-squared test. Statistically significant values (*P* < 0.05). Values in bold are statistically significant

### Multivariate analysis

Table 3 shows the results of the multivariate analysis of CDVA and UDVA. The calculated effects of the baseline confounders: visual acuity, mean keratometry, simulated cylinder, Sif, BCV, ThkMin and HOAs subtypes and the change in HOA subtypes, were given for both determinants. The confounders, CDVA and SIf at baseline were strongly related to a change in CDVA. An independent significant effect of the change in coma II was observed in the changes seen in CDVA (*P *= 0.048, β = 0.67). While no independent relationship between any changes in HOAs and UDVA were observed, astigmatism II at baseline was significantly associated with the change in UDVA (*P* = 0.03, β = -0.95).

**Table 3:** Multivariable analysis of the effect of a change in topographic parameters and optical aberrations on CDVA and UDVA 1 year after cross-linking

Δ CDVA		β Coefficient	[95% Conf. Interval]		*p* value
Baseline Confounder	CDVA	-0.33	-0.57	-0.09	0.012*
	Mean keratometry	0.02	0.00	0.05	0.061
	Simulated cylinder	-0.07	-0.22	0.08	0.328
	SIf	-0.07	-0.13	-0.01	0.027*
	BCV	-0.02	-0.07	0.03	0.448
	ThkMin	0.00	0.00	0.00	0.054
	Z (3, ± 3) trefoil	0.02	-0.15	0.20	0.786
	Z (3, ± 1) coma	0.08	-0.03	0.19	0.156
	Z (4, 0) spherical aberration I	0.15	-0.09	0.39	0.204
	Z (4, ± 2) astigmatism II	0.13	-0.25	0.51	0.454
	Z (5, ± 3) trefoil II	0.41	-0.56	1.38	0.372
	Z (5, ± 1) coma II	0.02	-0.83	0.87	0.964
Change in HOAs Subtype	Δ Z (3, ± 1) coma	-0.06	-0.18	0.07	0.334
	Δ Z (5, ± 1) coma II	0.67	0.01	1.34	0.0480*
	Δ Z (3, ± 3) trefoil	0.08	-0.17	0.34	0.488
	Δ Z (5, ± 3) trefoil II	-0.10	-0.95	0.74	0.795
	Δ Z (4, 0) Spherical Aberration I	0.08	-0.21	0.37	0.559
Δ UDVA		β Coefficient	[95% Conf. Interval]		*p* value
Baseline Confounder	UDVA	-0.19	-0.46	0.08	0.151
	Mean keratometry	0.03	-0.03	0.09	0.258
	Sim Cylinder	-0.20	-0.57	0.17	0.254
	SIf	-0.03	-0.16	0.10	0.611
	BCV	0.00	-0.13	0.12	0.942
	ThkMin	0.00	-0.01	0.00	0.388
	Z (3, ± 3) trefoil	0.03	-0.41	0.47	0.887
	Z (3, ± 1) coma	0.00	-0.26	0.27	0.986
	Z (4, 0) Spherical Aberration I	0.18	-0.31	0.68	0.425
	Z (4, ± 2) astigmatism II	-0.95	-1.78	-0.11	0.03*
	(5, ± 3) trefoil II	1.57	-0.70	3.85	0.153
	(5, ± 1) coma II	-0.46	-2.42	1.50	0.611
Change in HOAs Subtype	Δ Z (3, ± 1) coma	-0.16	-0.47	0.15	0.285
	Δ Z (5, ± 1) coma II	0.32	-1.32	1.96	0.676
	Δ Z (3, ± 3) trefoil	-0.23	-0.75	0.29	0.349
	Δ Z (5, ± 3) trefoil II	1.09	-0.97	3.16	0.266
	Δ Z (4, 0) Spherical Aberration I	-0.02	-0.71	0.67	0.947

Δ Changes in variables after cross-linking, *CDVA* Corrected distance visual acuity, *CI* Confidence interval, *HOA* Higher-order aberration, *UDVA* Uncorrected distance visual acuity, *SIf* Symmetry index front, *BCV* Baiocchi-Calossi-Versaci, *ThkMin* Minimum corneal thickness, *Z* Zernike, *HOAs* Higher-order aberrations, *UDVA* Uncorrected distance visual acuity

^*^ Statistically significant

## Discussion

Although corneal cross-linking was first introduced to halt the progression of KC, it has also been found to improve visual acuity and corneal topography characteristics [7, 8, 17]. Several studies have found increased higher-order aberrations in keratoconic corneas [18, 19], suggesting that such eyes may have poorer retinal images in comparison to normal eyes, leading to reduced visual acuity [4]. This study was designed to report on HOAs 12 months following A-CXL performed to treat KC, and to determine whether changes in HOAs are associated with a change in visual acuity.

Consistent with previously reported studies [7, 8, 20], this study showed significant improvements in MRSE, UDVA and CDVA at one year after cross-linking. The factors responsible for improving visual acuity after cross-linking are not yet clear. Kirgiz et al. postulated that anterior corneal flattening and posterior corneal steepening after cross-linking are key contributing factors in the stabilization of keratometric values and improvement of visual outcomes [21].

In this analysis, the Sirius Scheimpflug-Placido tomographer was used to measure the anterior corneal aberrations. Considering the finding that the anterior corneal surface contributes to approximately one half of the total aberrations of the eye [22], we opted to evaluate changes in corneal HOAs after CXL treatment rather than measuring whole-eye HOAs. The repeatability and reliability of anterior corneal aberrations with the Sirius is high [22]. Furthermore, particular attention was given to the anterior corneal HOAs, which are most relevant to clinical practice such as coma, trefoil, and spherical aberration [4].

Our results showed a significant sustained improvement in trefoil, secondary trefoil, secondary coma and secondary astigmatism (*P* < 0.05 for all) 12 months after CXL. We found similarities in our findings to those reported by other studies. Ghanem et al. reported a significant decrease in coma, trefoil, secondary astigmatism, secondary coma and secondary trefoil 2 years after CXL. The improvement in HOAs in KC eyes was attributed to the flattening of the corneal apex caused by the effect of CXL [23]. In this study, the significant flattening in the apical keratometry may explain the significant improvement in HOAs after CXL. However, in our previously reported study that compared an A-CXL irradiation protocol (10 mW/cm^2^ for 9 min) with the standard Dresden protocol (3 mW/ cm^2^ for 30 min), the standard protocol resulted in significantly greater anterior corneal flattening than the accelerated protocol [10]. Furthermore, we found that the impact of each treatment on the anterior HOAs was different; anterior trefoil was significantly decreased in the A-CXL group, whereas anterior total HOAs and coma were significantly decreased in the standard CXL group. Wisse et al. reported significant improvement in spherical aberrations 1 year after standard CXL [13]. Mazzotta et al. found significant improvement in coma 5 years after accelerated CXL (9mW/ cm^2^ for 10 min) [17]. Caporossi et al. reported a statistically significant reduction in coma aberrations between preoperative and 1-month postoperative values with a sustained effect 4 years after standard CXL [24]. However, no significant change in spherical aberration was established. These findings are consistent with this study, where no statistically significant difference in mean spherical aberrations values at one year after CXL was found. Greenstein et al. reported a significant improvement in total HOAs and coma when derived from the cornea alone as well as when measured as total ocular aberrations 1 year after standard CXL. Moreover, total corneal HOAs worsened by more than 1.0 µm only in one eye (out of 31 keratoconus eyes) [24]. In contrast, in this study although a statistically significant difference was not identified in total anterior HOAs, none of the 32 eyes showed an increase of 1.0 µm or more at 12 months follow-up.

Previous studies have demonstrated that the Zernike polynomials have a different impact on visual function [4]. Wisse et al. found that horizontal coma had the strongest relationship with change in UDVA [13]. In contrast, Greenstein et al. found no significant correlation between improvement in HOAs values and improvement in UDVA and CDVA after CXL [25]. Ghanem et al. found no correlations between changes in individual corneal aberrations and visual acuity after CXL [23]. On the contrary, our multivariate analysis demonstrated that coma II had the strongest relationship with changes in CDVA. Interestingly, no correlation was observed between HOAs subtypes changes and change in UDVA.

### Limitations

Several limitations of the study may have affected the results. The retrospective nature of the study is designed to analyse pre-existing data and is subject to bias. In addition, we were unable to evaluate total ocular HOAs as a wavefront device was not used in this study. We used the Sirius Scheimpflug tomography software algorithm, which calculates optical aberrations based on elevation maps, as opposed to an optical aberrometer. Another limitation in our study is that eight of the patients had both of their eyes analysed which could be a source of statistical bias. As corneal biomechanical changes continue for longer than one year after corneal cross-linking [26, 27], the relatively short follow-up time is another limitation of this study. The power of the study is influenced by several factors, but as a general rule, higher power is obtained by increasing the sample size. Using small sample sizes may yield unreliable results. Increasing the sample size and a power calculation in future studies will be key to limit the risk of sampling bias. Further studies with a larger sample size and longer follow-up period to evaluate wavefront aberrations in addition to Scheimpflug-based aberrations are recommended.

## Conclusion

This study demonstrated that anterior corneal higher-order aberrations, in particular trefoil, trefoil II, coma II and astigmatism II, improved after a 9-min protocol of accelerated corneal cross-linking. Only changes in coma II had a significant and independent effect on corrected distance visual acuity.

## Data Availability

The datasets generated and analysed during the current study are not publicly available due their containing information that could compromise the privacy of research participants but are available from the corresponding author (AS) on reasonable request.

## References

[1] Rabinowitz YS. Keratoconus. Surv Ophthalmol. 1998 Jan-Feb;42(4):297–319. https://pubmed.ncbi.nlm.nih.gov/9493273/10.1016/s0039-6257(97)00119-7 9493273

[2] Davidson AE, Hayes S, Hardcastle AJ, Tuft SJ (2014). The pathogenesis of keratoconus. Eye (Lond).

[3] Blackburn BJ, Jenkins MW, Rollins AM, Dupps WJ (2019). A review of structural and biomechanical changes in the cornea in aging, disease, and photochemical crosslinking. Front Bioeng Biotechnol.

[4] Jinabhai A, Radhakrishnan H, O’donnell C (2009). Higher order aberrations in keratoconus: a review. Optometry Practice.

[5] Wollensak G, Spoerl E, Seiler T (2003). Riboflavin/ultraviolet-a-induced collagen crosslinking for the treatment of keratoconus. Am J Ophthalmol.

[6] Arbelaez MC, Sekito MB, Vidal C, Choudhury SR (2009). Collagen cross-linking with riboflavin and ultraviolet-A light in keratoconus: One-year results. Oman J Ophthalmol.

[7] Artieda J, Mahíllo-Fernández I, Morote I, Alba N (2020). Analysis of visual, refractive, topographic and aberrometric changes in different uncommon accelerated cross-linking protocols in keratoconus: a 12 month follow-up. J EuCornea.

[8] Kirgiz A, Eliacik M, Yildirim Y (2019). Different accelerated corneal collagen cross-linking treatment modalities in progressive keratoconus. Eye Vis (Lond).

[9] Singal N, Ong Tone S, Stein R, Bujak MC, Chan CC, Chew HF, El-Defrawy S, Jin Y, Kranemann C, Rabinovitch T, Rootman DS, Slomovic AR, Cohen A, Dai D, Hatch W (2020). Comparison of accelerated CXL alone, accelerated CXL-ICRS, and accelerated CXL-TG-PRK in progressive keratoconus and other corneal ectasias. J Cataract Refract Surg.

[10] Salman AM, Darwish TR, Haddad YH, Shabaan RH, Askar MZ (2021). Accelerated versus standard corneal cross-linking for progressive keratoconus in Syria. J Ophthalmic Vis Res.

[11] Gomes JA, Tan D, Rapuano CJ, Belin MW, Ambrósio R, Guell JL, Malecaze F, Nishida K, Sangwan VS, Group of Panelists for the Global Delphi Panel of Keratoconus and Ectatic Diseases (2015). Global consensus on keratoconus and ectatic diseases. Cornea.

[12] Hersh PS, Greenstein SA, Fry KL (2011). Corneal collagen crosslinking for keratoconus and corneal ectasia: One-year results. J Cataract Refract Surg.

[13] Wisse RP, Gadiot S, Soeters N, Godefrooij DA, Imhof SM, van der Lelij A (2016). Higher-order aberrations 1 year after corneal collagen crosslinking for keratoconus and their independent effect on visual acuity. J Cataract Refract Surg.

[14] Amsler M (1946). Kératocõne classique et kératocône fruste; arguments unitaires [Classic keratocene and crude keratocene; Unitary arguments]. Ophthalmologica..

[15] Salman A, Darwish T, Ghabra M, Kailani O, Khalil H, Shaaban R (2021). Clinical Outcomes of Accelerated Corneal Cross-Linking for Pediatric Keratoconus. J Ophthalmol.

[16] International Organization for Standardization (ISO). Ophthalmic Optics and Instruments-Reporting Aberrations of the Human Eye. Geneva, Switzerland, ISO, 2008: (ISO 24157:2008). https://www.iso.org/standard/42041.html

[17] Mazzotta C, Raiskup F, Hafezi F, Torres-Netto EA, ArmiaBalamoun A, Giannaccare G, Bagaglia SA (2021). Long term results of accelerated 9 mW corneal crosslinking for early progressive keratoconus: the Siena Eye-Cross Study 2. Eye Vis (Lond).

[18] Alió JL, Shabayek MH (2006). Corneal higher order aberrations: a method to grade keratoconus. J Refract Surg.

[19] Shneor E, Piñero DP, Doron R (2021). Contrast sensitivity and higher-order aberrations in Keratoconus subjects. Sci Rep.

[20] Greenstein SA, Fry KL, Hersh PS (2011). Corneal topography indices after corneal collagen crosslinking for keratoconus and corneal ectasia: one-year results. J Cataract Refract Surg.

[21] Kırgız A, Atalay K, Çabuk KŞ, Kaldırım H, Taşkapılı M (2016). Factors affecting visual acuity after accelerated crosslinking in patients with progressive keratoconus. Arq Bras Oftalmol.

[22] Bayhan HA, AslanBayhan S, Muhafız E, Can I (2014). Repeatability of aberrometric measurements in normal and keratoconus eyes using a new Scheimpflug-Placido topographer. J Cataract Refract Surg.

[23] Ghanem RC, Santhiago MR, Berti T, Netto MV, Ghanem VC (2014). Topographic, corneal wavefront, and refractive outcomes 2 years after collagen crosslinking for progressive keratoconus. Cornea.

[24] Caporossi A, Mazzotta C, Baiocchi S, Caporossi T (2010). Long-term results of riboflavin ultraviolet a corneal collagen cross-linking for keratoconus in Italy: the Siena eye cross study. Am J Ophthalmol.

[25] Greenstein SA, Fry KL, Hersh MJ, Hersh PS (2012). Higher-order aberrations after corneal collagen crosslinking for keratoconus and corneal ectasia. J Cataract Refract Surg.

[26] Salman A, Ali A, Rafea S, Omran R, Kubaisi B, Ghabra M, Darwish T (2022). Long-term visual, anterior and posterior corneal changes after crosslinking for progressive keratoconus. Eur J Ophthalmol.

[27] Raiskup-Wolf F, Hoyer A, Spoerl E, Pillunat LE (2008). Collagen crosslinking with riboflavin and ultraviolet-A light in keratoconus: long-term results. J Cataract Refract Surg.

